# Current Nutritional Approaches to Maintaining and Improving Broiler Meat Quality: Part 1, Diet Manipulation and Feeding Management

**DOI:** 10.3390/ani16203194

**Published:** 2026-10-12

**Authors:** Tarek A. Ebeid, Mohammed Aladhadh, Abdullah N. Alkhalaf

**Affiliations:** 1 Department of Animal and Poultry Production, College of Agriculture and Food, Qassim University, P.O. Box 6622, Buraidah 51452, Saudi Arabia; 2 Department of Food Science and Human Nutrition, College of Agriculture and Food, Qassim University, Buraydah 51452, Saudi Arabia; 3 Department of Pathology & Lab. Diagnosis, College of Veterinary Medicine, Qassim University, Buraydah 51452, Saudi Arabia

**Keywords:** chickens, meat, low-protein diets, low-energy diets, fats, omega-3 enrichment, oxidative stability, breast myopathies, feed restriction, pre-slaughter fasting

## Abstract

Global poultry meat consumption is expected to increase by about 29 million tons (20%) between 2026 and 2035, making poultry the largest contributor to global meat-consumption growth. Total global meat consumption is projected to reach approximately 412 million tons by 2035, representing a 12% increase over the base period. Commercial broiler production is characterized by fast growth and great feed efficiency, leading to increased concerns about meat quality because fast-growing breeds often exhibit poorer quality traits. The objective of the present review is to investigate the role of recent nutritional strategies, specifically diet manipulation and feeding regimes and management, in improving or maintaining the physicochemical and nutritional properties of broiler meat. A dietary crude protein reduction by −1.5% to −3.0% that is balanced with essential amino acids might maintain meat physicochemical characteristics. Dietary lipid sources (such as vegetable oils, tallow, and insect fats) with/without emulsifiers modified the fatty acid profile in meat according to the source used; pH was generally unaffected, whereas color and texture traits showed source- and dose-dependent responses, ranging from no effect (e.g., olive pomace acid oil) to altered lightness, redness, or shear force (e.g., soybean oil, rice bran oil, high-level insect fat inclusion). Furthermore, the dietary inclusion of omega-3 sources like fish oil, linseed products, and marine algae effectively enriched meat with n-3 polyunsaturated fatty acids. Dietary marine algae provided beneficial antioxidants that help in maintaining meat oxidative stability via reducing lipid peroxidation, leading to a prolonged shelf life. The addition of carbohydrases or multi-enzyme cocktails to a low-energy diet or low-density feed ingredients helped in maintaining the physical properties of broiler meat. Feed restriction programs had no negative impacts on physical meat quality parameters, but they were effective tools for mitigating breast muscle myopathies like wooden breast, white striping, and spaghetti meat. Optimizing pre-slaughter fasting to a moderate duration (8–12 h) is essential to prevent stress-induced meat quality degradation. Collectively, these findings indicate that the reviewed nutritional approaches can improve or maintain broiler meat quality; reduced dietary crude protein additionally lowers nitrogen excretion, a mechanism plausibly linked to reduced ammonia emissions and feed cost, although direct economic or environmental impact data were not evaluated in this review.

## Simple Summary

Poultry meat is an important source of food worldwide, and its consumption is expected to continue increasing. However, the very rapid growth of modern broiler chickens can negatively affect some aspects of meat quality and increase the occurrence of breast muscle problems. This review examines recent nutritional and feeding strategies that can help maintain or improve the quality and nutritional value of broiler meat. Reducing dietary protein while providing sufficient essential amino acids can maintain desirable meat quality and may reduce feeding costs. Different dietary fat sources can modify the fatty acid profile of meat; most sources leave pH largely unaffected, although some (e.g., soybean oil, rice bran oil) alter color and texture traits such as lightness, redness, or shear force depending on the specific oil and inclusion level used. Omega-3-rich ingredients such as fish oil, linseed products, and marine algae can increase beneficial omega-3 fatty acids in meat, generally with limited effects on pH and color, though higher inclusion levels of some sources (e.g., DHA-rich microalgae) can increase drip loss and shear force. Enzyme supplementation can help maintain meat quality when lower-energy diets or less digestible feed ingredients are used. Feed restriction can help reduce breast muscle problems and, in most of the studies reviewed, did not adversely affect major physical meat quality traits. In addition, a moderate pre-slaughter fasting of approximately 8–12 h may help minimize the stress-related deterioration of meat quality. Overall, appropriate nutritional and feeding strategies can support meat quality and may contribute to the production of nutritionally valuable meat for consumers.

## 1. Introduction

Globally, there has been an increase in consumer demand for poultry meat because of its greater cost-effectiveness, as well as its low price and no religious and social issues [1]. It is consumed as fresh, frozen, canned and processed products such as sausages and nuggets. According to the most recent OECD-FAO Agricultural Outlook 2026–2035 [2], global poultry meat consumption is projected to remain the largest single contributor to worldwide meat-consumption growth over the coming decade, increasing by approximately 29 million tons (+20%) over the 2026–2035 outlook period, against a projected total global meat consumption of approximately 412 million tons (retail weight equivalent) by 2035—a 12% increase over the base period. Global poultry meat production is projected to reach approximately 146 million tons by year, of which chicken meat is expected to account for the large majority, approximately 90% [3]. Broilers—meat-type chickens raised specifically for meat production, as distinct from a genetic breed—represent the predominant chicken meat production category, with specialized, intensive broiler production systems estimated to contribute approximately 93% of global chicken meat output, as opposed to spent layers or backyard/extensive systems [3]. This increase is a result of commercial broiler chickens’ rapid growth, superior feed conversion efficacy, short production cycles, and adaptability to diverse production systems [4]. However, there have been some recent concerns expressed about the quality of meat because fast-growing broilers often exhibit poorer meat quality than slow-growing broilers [5]. It is well identified that several factors have a direct impact on meat quality, including breed, sex, age, nutrition, housing, management, environmental temperature, stress, processing, and storage [6].

It should be noted that the primary studies discussed in this review were conducted using different broiler genetic strains (e.g., Ross 308, Cobb 500, Arbor Acres), sexes, and ages/body weights at slaughter (ranging from approximately 21 to ≥42 d of age), as well as, in a limited number of cases, slow-growing or dual-purpose genotypes rather than fast-growing commercial broilers (specified where applicable, e.g., Section 5.1). Because genetic strain, sex, and age at slaughter are themselves well-established determinants of muscle fiber characteristics, growth rate, and postmortem metabolism [5], part of the variability observed between studies employing the same nutritional strategy may be attributable to these zootechnical factors rather than to the dietary treatment alone.

**Figure 1:**
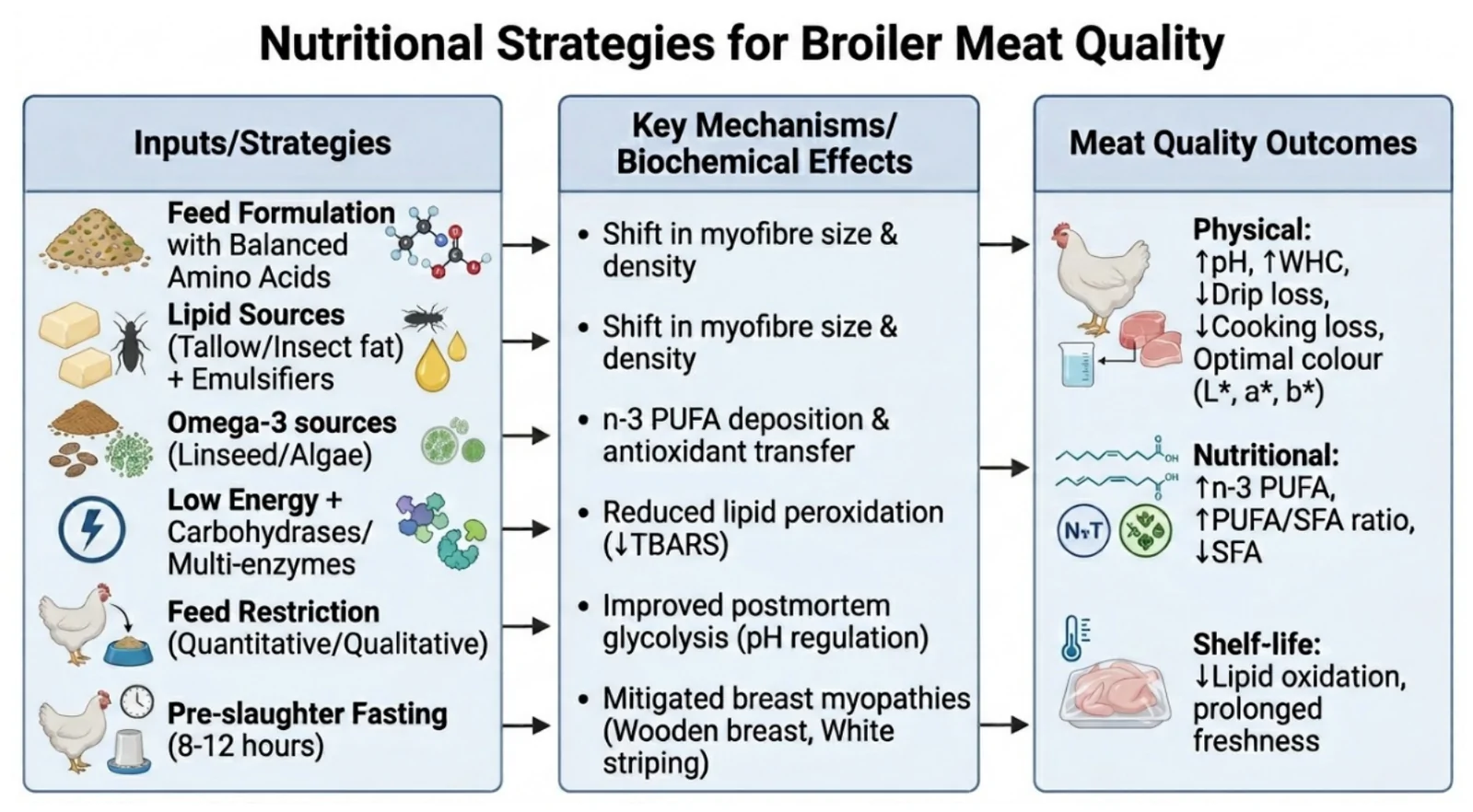
A summary of the impacts of diet manipulation and feeding management on meat quality traits in broilers; PUFAs = polyunsaturated fatty acid; TBARS = thiobarbituric acid reactive substances; WHC = water-holding capacity; L* = lightness; a* = redness; b* = yellowness; SFAs = saturated fatty acids (↑: Increase and ↓: Decrease).

Several recent reviews have addressed related aspects of the nutritional regulation of broiler meat quality. Choi et al. [7] examined nutritional strategies for improving meat quality and composition specifically under heat stress and other production-challenge conditions, with emphasis on antioxidants, vitamins, and trace minerals rather than the protein-, lipid-, energy-, and management-based strategies addressed here. A further review focused specifically on low-crude-protein diets, covering their effects on gene expression, sustainability, and greenhouse gas emissions in broiler production [8], overlapping with Section 3 of the present review but not extending to lipid/omega-3 sources, exogenous enzymes, feed restriction, or pre-slaughter fasting. Most recently, Niu et al. [9] reviewed the nutritional regulation of poultry meat quality from a phenotype-to-mechanism perspective, centering on protein, amino acid, and lipid regulation at the molecular level across poultry species broadly. The present review extends and updates two previous focused reviews: Ebeid et al. [10], which examined the effects of feed restriction on broiler meat quality, and Ebeid et al. [11], which reviewed dietary exogenous enzyme supplementation in broilers. The present review incorporates the primary literature published subsequent to both reviews and situates each topic within the broader six-strategy diet manipulation framework outlined below, rather than treating either strategy as a stand-alone subject. In contrast to these works, the present review (i) is restricted to broilers; (ii) integrates six nutrition- and management-based strategies (crude protein/amino acids, lipid sources and emulsifiers, omega-3 enrichment, energy/enzyme supplementation, feed restriction, and pre-slaughter fasting) within a single, physicochemically and nutritionally organized framework; and (iii) explicitly separates diet composition strategies (Part 1) from functional feed additive strategies (Part 2, companion review), a scope broader than that of any single one of the works cited above. Therefore, the objective of the present review (Part 1) is to emphasize the recent results linked to the role of diet manipulation and feeding patterns—namely crude protein and amino acid balancing, dietary lipid and omega-3 sources, exogenous enzyme supplementation, feed restriction, and pre-slaughter fasting—in improving or maintaining meat quality characteristics and its nutritional value (Figure 1). A companion review (Part 2) will separately address the role of functional feed additives (e.g., phytogenics, organic acids, probiotics, prebiotics, and antioxidant/vitamin–mineral supplements) in modulating broiler meat quality, reflecting a deliberate division between diet/feeding-regime-based strategies (Part 1) and additive-based strategies (Part 2).

## 2. Literature Search Strategy

A structured literature search was conducted to identify relevant scientific publications addressing nutritional and feeding management strategies affecting broiler meat quality. The literature search was performed using PubMed, Scopus, Web of Science, and Google Scholar. The search focused primarily on recent peer-reviewed studies published during the last five years, while selected earlier publications were retained when they provided important foundational information or established concepts relevant to the topic. Combinations of keywords and Boolean operators were used, including broiler chickens, poultry meat quality, dietary manipulation, crude protein, amino acids, dietary fat, omega-3 fatty acids, fish oil, linseed, microalgae, enzymes, low-energy diets, feed restriction, feeding management, pre-slaughter fasting, physicochemical properties, meat composition, and breast muscle myopathies. Retrieved publications were initially screened based on their titles and abstracts, followed by full-text assessment when considered relevant to the scope of this review. Priority was given to original peer-reviewed studies that directly investigated the effects of dietary composition, feed restriction, feeding regimes, or pre-slaughter management on physicochemical characteristics, chemical composition, fatty acid profile, sensory attributes, oxidative stability, or breast muscle abnormalities in broiler chickens. Studies that were not directly related to broiler meat quality or did not provide relevant nutritional or feeding management information were excluded. Particular emphasis was placed on recent studies to reflect current developments in poultry nutrition, whereas older studies were included when required to provide fundamental or supporting information. A total of 77 scientific journal articles were included in the present review. In addition, two authoritative sources from FAO and OECD/FAO were consulted to provide current information on global poultry production and consumption, resulting in a total of 79 references cited in this manuscript. The selected literature was critically evaluated and synthesized according to the major topics addressed in this review, including dietary crude protein and amino acids, dietary fats and emulsifiers, omega-3 fatty acids, energy and enzyme supplementation, feed restriction, and pre-slaughter fasting.

## 3. Physicochemical Properties of Meat

The physicochemical properties of meat are important factors of its quality, palatability, and consumer acceptance. These properties include pH, water-holding capacity (WHC), color, tenderness, drip loss, cooking loss, shear force, and chemical composition such as moisture, crude protein (CP), fat (ether extract, EE), and ash contents. These properties influence meat storage stability, processing performance, and consumer decisions regarding meat quality and meat products [6]. The ultimate pH of meat, which results from postmortem glycolysis and lactic acid accumulation, profoundly affects other quality traits, whereas low pH often results in pale, soft, and exudative meat, while high pH produces dark, firm, and dry meat [12]. Meat’s pH value has a direct impact on meat quality characteristics such as color, tenderness, WHC, juiciness, and shelf life [6]. Low pH (below 5.8) decreases WHC and increases drip and cooking losses, ultimately resulting in the loss of moisture, nutrients, and flavor. Meat color (myoglobin content) has a significant impact on consumers’ propensity to buy, making it one of the most significant properties of meat quality because people tend to correlate the appearance of meat with its freshness. Poultry meat color [involving lightness (L*), redness (a*), and yellowness (b*) values] depends on postmortem temperature sarcoplasmic protein denaturation and WHC, pre-slaughter stress, and pH [13]. WHC influences meat color, tenderness, juiciness, drip loss, cooking loss, and yield during processing and cooking [14]. Indeed, alterations in pH values affect WHC, whereby greater pH values increase WHC and consequently enhance the juiciness and tenderness of meat [15]. Tenderness, one of the most important sensory attributes, is influenced by muscle fiber structure, collagen content, and postmortem proteolysis [16]. The major factors determining meat tenderness are the stage of development of the connective tissue and myofibrillar proteins’ contractile condition and the rate of rigor mortis development [17]. Sensory traits (cohesiveness, hardness, juiciness, chewiness, fibrosity, and flavor) are important for consumers’ preferences [18]. Meat flavor consists of a complex blend of volatile chemicals produced through thermally induced reactions of non-volatile components. The exclusive flavor and aroma of chicken meat are linked to lipid-derived chemicals (such as hexenal, heptenal, octenal, nonenal, undecenal, and dodecenal) and aldehydes (such as octanal, nonanal, decanal, and dodecadienal) [19]. Collectively, these physicochemical traits determine the overall eating quality and consumer perception of broiler meat [20].

Meat chemical composition consists of water, protein, fat, minerals, and vitamins. The approximate composition of broiler chicken meat varies according to meat cut, tissue type, and other factors, but poultry meat is generally characterized by a high moisture and protein content and relatively low fat content in lean cuts [21]. From the perspective of nutrition, poultry meat is considered a nutritious and healthy food because it offers high-quality protein comprising all essential amino acids, vitamins (niacin, vitamin B6, and vitamin B12), and minerals (phosphorus, selenium, and zinc) [22,23]. In addition, poultry meat has low energy, fat, and cholesterol contents, specifically when eaten without skin. Moreover, it is rich in desirable polyunsaturated fatty acids (PUFAs) and monounsaturated fatty acids (MUFAs) and low in saturated fatty acids (SFAs), making this meat accessible to all segments of people [24]. Several bioactive substances, including glutathione, L-carnitine, taurine, anserine, creatine, and carnosine, are also present in poultry meat, and most of them can be enhanced by proper nutrition [25]. Compared with red and processed meats, poultry meat consumption is associated with a lower relative risk of certain cancers and cardiovascular disease, based on epidemiological evidence [26,27].

## 4. Diet Manipulation Influencing Broiler Meat Quality

### 4.1. Crude Protein and Amino Acids

There is an intense trend towards reducing the CP content of feed and compensating for this by adding amino acids to diminish the feed cost and also reduce the rate of nitrogen excretion in litter and, consequently, the emission of ammonia in poultry houses, which have a positive effect on the environment [28,29,30]; a broader treatment of low-CP diets and their sustainability implications is provided by Salahi et al. [8], while the present section focuses specifically on meat physicochemical and chemical composition outcomes. Ma et al. [31] reported that reducing dietary CP by −3% in male Cobb broilers during the 42 d production period increased breast meat shear force and altered several physicochemical characteristics, including pH_24h_, the L* value, the a* value, and drip loss. However, the supplementation of a low-CP diet with 0.13 or 0.26% isoleucine reduced shear force and improved several of these responses toward the control values, indicating that the meat quality response was influenced not only by the magnitude of CP reduction but also by the adequacy of the limiting amino acid supply. The study also showed that isoleucine supplementation modified muscle fiber characteristics, providing a potential biological explanation for the changes in tenderness. Moreover, it also demonstrated that dietary treatments reduced fat accumulation in broilers via the activation of 5′adenosine monophosphate-activated protein kinase and Janus kinase 2/signal transducer and activator of transcription 3 signaling pathways, suggesting that low dietary CP can directly regulate energy partitioning and lipogenesis [31]. Recently, Askri et al. [32] reported that reducing dietary CP content by −2.0% with the addition of 2.5% meat and bone meal in grower and finisher diets containing 20.4% and 19.5% CP, respectively, increased pH_24h_ and sarcoplasmic protein solubility and reduced the b* value and cooking loss, whereas the L* value, a* value, and Warner–Bratzler shear force were not affected in breast muscle. Brink et al. [28] observed that low-protein diets in the grower phase (17.5% vs. 18.7% and 20.5%) and in the finisher phase (16.6% vs. 18.0% and 19.5%) enhanced the L∗ value and increased thawing loss. They proposed that low-protein diets increased the size of myofibers and reduced their density, thereby altering light reflectance properties and increasing exudative losses. Enlarged myofibers generally possess lower capillary density and result in a paler meat appearance [33]. Similarly, Benahmed et al. [29] demonstrated that lowering CP by −1.5% to −3.0% by partially substituting corn and canola meal with soybean meal reduced the b* value, cooking loss, and Warner–Bratzler shear force in breast meat, which was in contrast with Ma et al. [31], who indicated an increase in shear force due to reducing CP by −3% with the supplementation of isoleucine (0.26%). These contrasting findings regarding shear force might be related to differences in broiler genetic strain, sex, dietary ingredient composition, the magnitude and duration of CP reduction, and age/body weight at slaughter between the two trials, in addition to the degree of connective tissue maturation and postmortem proteolytic activity, all of which are known to influence myofibrillar structure and tenderness independently of dietary protein level [5]. Several studies indicated that moderate CP reduction had a limited effect on meat pH, color, and cooking loss [30,34,35]. Son et al. [30] pointed out that −1% to −4% CP reductions during the grower phase (control containing 20% CP) and finisher phase (control containing 20% CP) had no negative impact on pH, the L* value, the b* value, cooking loss, or shear force; however, reducing CP by −1% enhanced the WHC level and increased the a* value in the breast meat of broilers. Duque-Ramírez et al. [35] reported that reducing CP content by −1.0% with the addition of protease (100–200 ppm) had no significant influence on the L* value, a* value, or cooking loss, while the b* value was significantly increased in breast meat. Wang et al. [34] elucidated that low-protein diets (−2.0%) enhanced the b* value and L* value; meanwhile, the a* value and pH were not affected in thigh muscle. They also observed that low-protein diets (−2.0%) had no significant effect on pH or the a* value, b* value or L* value in the breast meat of broilers. Collectively, it might be concluded that a moderate CP reduction by −1.5% to −3.0% accompanied by fortification with adequate essential amino acids might maintain meat physical quality characteristics under specific nutritional conditions, particularly when essential amino acid requirements are adequately met and the ingredient substitutions are appropriately formulated. Future research should focus on integrating metabolomics, postmortem proteolytic activity, and muscle histochemistry to elucidate the biological mechanisms of meat quality responses to low dietary CP.

**Figure 2:**
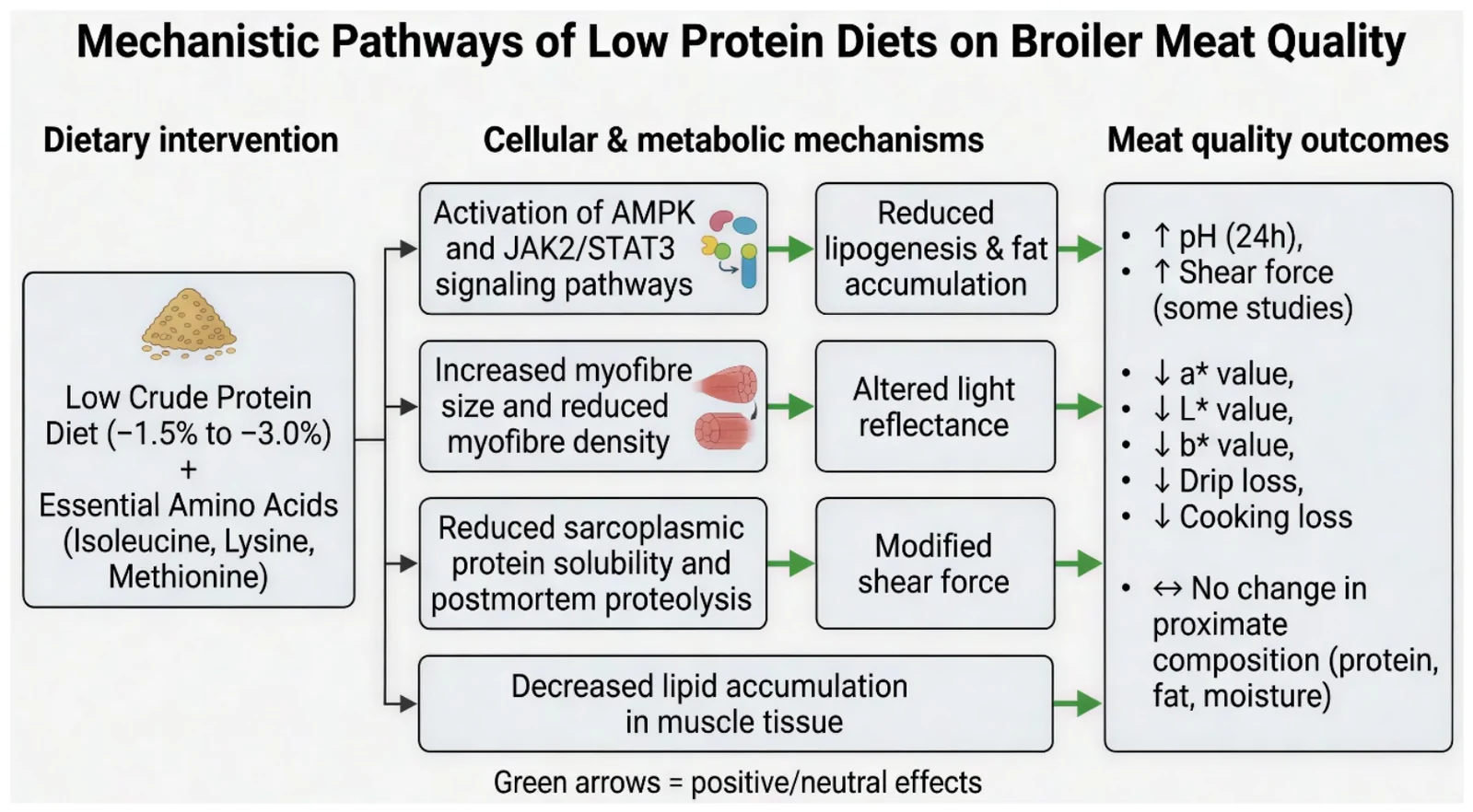
A conceptual diagram illustrating the mechanistic pathways by which low-crude-protein diets (−1.5% to −3.0%) supplemented with essential amino acids influence broiler meat quality (↑: Increase and ↓: Decrease).

Regarding meat chemical composition, a moderate CP reduction by −1.5% to −3.0% along with fortification with adequate essential amino acids had no detrimental effects on meat proximate composition [29,32]. Askri et al. [32] postulated that reducing dietary CP content by −2.0% with the addition of 2.5% meat and bone meal in grower and finisher diets containing 20.4% and 19.5% CP, respectively, had no significant impact on breast meat proximate composition (protein, lipids, and dry matter, “DM”). Similarly, Benahmed et al. [29] demonstrated that lowering CP by −1.5% to −3.0% by partially substituting corn and canola meal with soybean meal did not affect breast meat chemical composition, including protein, lipids, and DM. These results indicate that modern broiler chickens can maintain muscle protein accretion when fed a moderate low-CP diet fortified with adequate essential amino acids. However, Son et al. [30] noted that 1–4% CP reductions during the grower phase (control containing 20% CP) and finisher phase (control containing 20% CP) decreased moisture content and increased EE content in a dose-dependent manner, but CP and crude ash content were not affected in the breast meat of broilers. These findings suggest that CP reductions may be involved in shifting nutrient partitioning toward lipid deposition rather than lean tissue accretion. Importantly, Usturoi et al.’s [36] findings should not be interpreted as evidence of adverse effects of a −0.5% CP reduction because their study compared a standard diet with diets containing 0.5 percentage points more CP during the growing phase and 1.0 percentage point more CP during the finishing phase in slow-growing Hubbard JA757 broilers. Their findings therefore provide a different nutritional context and do not establish a threshold for the adverse effects of CP reduction. Taken together, the available evidence suggests that a moderate CP reduction of approximately −1.5% to −3.0% percentage points may be compatible with maintaining broiler meat chemical composition when essential amino acid requirements are adequately met; however, this range should be regarded as conditional rather than as a universal threshold applicable across genotypes, ages, dietary formulations, or production systems. Moreover, excessive CP reduction may impair meat protein content and increase lipid content (Table 1, Figure 2).

### 4.2. Fats and Emulsifiers

In poultry diets, dietary fats obtained from plant and animal sources play a significant role as energy sources. Studies indicated that different oil sources and byproduct oils can generally be incorporated into broiler diets without compromising pH, cooking loss, or chilling loss, although effects on color and texture vary by oil type [37,38]. Elbaz et al. [37] reported that a 3% dietary incorporation of different oil sources (soybean oil, corn oil, olive oil, or fish oil) had no negative impact on pH, cooking loss, or chilling loss in breast meat; however, soybean oil specifically increased shear force and the a* value while reducing the L* and b* values, indicating that not all oil sources are functionally equivalent with respect to color and tenderness outcomes in broilers reared under cyclic heat stress conditions. Also, Albendea et al. [38] compared three oil sources (palm oil, refined olive pomace oil, and olive pomace acid oil) and pointed out that dietary 6% olive pomace acid oil had no negative influence on meat color (L*, a*, or b* values) or sensory scores in breast meat in comparison with palm oil or refined olive pomace oil. However, Al-Abdullatif et al. [39] showed that the substitution of dietary 5% palm oil with 5% rice bran oil lowered pH_20min_, and pH_24h_, but increased cooking loss in breast and thigh muscles. Yin et al. [40] reported that dietary 5% soybean oil reduced drip loss in breast meat in comparison with lard, while pH_45min_, pH_24h_, and shear force were not affected. There is growing interest in using insects as alternative protein and fat sources in poultry nutrition because of their nutritional value and sustainability potential [41,42]. Popova et al. [43] pointed out that the dietary inclusion of low-fat *Tenebrio molitor* larva meal up to 5% had no harmful effect on physical meat parameters; however, high levels (7.5 and 10%) reduced pH_24h_ and the a* value but elevated the L* value, drip loss, and cooking loss in breast meat. Likewise, Aslam et al. [41] compared black soldier fly larva fat, included as the sole supplemental dietary fat source at 34.0 g/kg (3.4%) in the starter diet (1–14 d) and 51.5 g/kg (~5.2%) in the grower diet (15–35 d), with equivalent inclusion levels of different lipid sources (soybean oil, rapeseed oil, palm oil, palm kernel fatty acid distillate, poultry fat, pig lard, and beef tallow) and reported that substituting soybean oil with black soldier fly larva fat had no significant impact on the pH at slaughter, pH_20min_, pH_2h_, pH_24h_, color (L*, a*, or b* values), or drip loss of broiler breast meat. However, the addition of black soldier fly larva fat increased drip loss after 48 h compared with poultry fat, pig lard, beef tallow, rapeseed oil, and palm oil. Such findings emphasize that the effects of lipid sources on meat physical characteristics depend largely on their fatty acid profile and their influence on cell membrane stability and myoglobin oxidation. Currently, exogenous emulsifiers have gained interest because they improve fat digestion and energy retention, leading to enhanced growth performance, carcass traits, and meat quality [44,45,46]. Several studies demonstrated that dietary emulsifiers and lipase maintained normal meat physical quality traits in broilers [44,45,47]. Tenório et al. [44] studied the effect of two fat sources (acid soybean oil or degummed soybean oil) with or without emulsifier (350 g/ton glyceryl polyethylene glycol ricinoleate) and showed that dietary degummed soybean oil increased WHC, shear force, and the b* value but reduced pH_24h_ in comparison with acid soybean oil. The addition of emulsifier to diets did not provide extra benefits to the meat quality characteristics of breast meat. Similarly, Liu et al. [47] elucidated that adding an emulsifier mixture (0.05% sodium stearoyl-2-lactylate and 0.10% 1, 3-diacylglycerol) to a low-energy diet (reduced by −100 kcal/kg) had no effect on pH, color (L*, a*, and b* values), WHC, cooking loss, or drip loss in breast muscle. Ahmad et al. [45] confirmed that the dietary addition of lipase (0.04%) and/or lysolecithin (0.04%) to a low-energy diet (reduced by −100 kcal/kg) had no negative effect on pH_2h_, pH_24h_, cooking loss, drip loss, or WHC in breast and thigh muscles. The limited improvements observed in previous studies may be partly attributed to the relatively efficient endogenous digestion and absorption of dietary lipids in healthy broilers under non-challenging conditions. Consequently, supplementation with exogenous emulsifiers or lipolytic enzymes may provide limited additional benefits when basal dietary fat digestion is already sufficient to meet the birds’ physiological requirements. Their effects may become more evident when lipid digestion or nutrient utilization is compromised, such as under low-energy diets, with poorly digestible fat sources, or during physiological stress. On the other hand, Shen et al. [48] proved that dietary lecithin supplementation (1 g/kg) increased the L* value and drip loss but reduced shear force, while it had no significant influence on pH, the a* value or the b* value in breast meat. Moreover, Eid et al. [46] noted that the dietary inclusion of 0.5 and 1 g/kg of lysophospholipid emulsifiers increased pH, WHC, and tenderness in broilers exposed to oxidative stress, indicating that dietary emulsifiers may exert greater efficiency during physiological challenges. Taken together, the effects of alternative lipid sources on breast meat physical quality are source- and dose-dependent rather than uniformly neutral. Palm oil, refined olive pomace oil, and olive pomace acid oil showed no detectable effect on color or sensory scores [38]. Rice bran oil, by contrast, lowered pH_20min_ and pH_24h_ and increased cooking loss relative to palm oil [39], with drip loss increasing again after 48 h relative to poultry fat, lard, tallow, rapeseed oil, and palm oil at higher inclusion [41]. Exogenous emulsifiers generally provided no additional benefit to meat quality under standard dietary conditions [44,45,47] but improved pH, WHC, and tenderness specifically when broilers were exposed to oxidative stress [46], indicating that emulsifier efficacy is condition-dependent rather than a general property of these additives.

With respect to meat chemical composition, Al-Abdullatif et al. [39] showed that the substitution of dietary 5% palm oil with 5% rice bran oil reduced CP, increased EE and caused no alteration in moisture or ash in breast and thigh meats. They also indicated that dietary rice bran oil instead of palm oil increased α-linolenic acid (C18:3 n3) and n-3PUFAs, and reduced the n-6/n-3 PUFA ratio and SFAs in breast and thigh muscles, which offer a positive impact on human health. Similarly, Albendea et al. [38] pointed out that dietary 6% olive pomace acid oil increased MUFAs, α-linolenic acid (C18:3 n3), and n-3 PUFA but reduced α-tocopherol, while the thiobarbituric acid reactive substances (TBARS) value was not affected in breast meat in comparison with palm oil or refined olive pomace oil. Also, Popova et al. [43] pointed out that the dietary inclusion of low-fat *Tenebrio molitor* larva meal up to 5% had no harmful effect on meat chemical composition; however, high levels (7.5 and 10%) reduced the CP, EE, and moisture of breast meat. They also indicated that increasing the dietary concentration of low-fat *Tenebrio molitor* larva meal increased SFAs and MUFAs while reducing PUFA content in breast and thigh meats. Aslam et al. [41] postulated that substituting soybean oil with black soldier fly larva fat enriched breast meat content with n-3PUFAs and α-linolenic acid (C18:3 n3), while it had no significant effect on SFAs, UFAs, MUFAs, PUFAs, or the PUFA/SFA ratio in breast meat in comparison with soybean oil.

Some studies demonstrated that dietary emulsifiers and lipase did not significantly alter total lipid content or the fatty acid profile in breast muscle in broilers [45,49]. Saleh et al. [49] posited that the addition of an exogenous emulsifier blend (phosphatidyl choline, lysophosphatidyl choline, and polyethylene glycol ricinoleate, 500 g/ton) to a low-energy diet (reduced by −50 kcal/kg) had no significant effect on the total lipid, α-tocopherol, oleic acid (C18:1), linoleic acid (C18:2), or α-linolenic acid (C18:3 n3) content of breast muscle. Ahmad et al. [45] confirmed that the dietary addition of lipase (0.04%) and/or lysolecithin (0.04%) to a low-energy diet (reduced by −100 kcal/kg) had no negative effect on the fatty acid profile in breast muscle. In summary, the meat lipid profile is a mirror of the fatty acid content of diets. Also, lipid digestibility, metabolism, and oxidative stability play a significant role in modifying meat chemical composition. The dietary addition of emulsifiers did not provide additional benefits to meat chemical composition; however, further studies are required to clarify their long-term effects (Table 2).

### 4.3. Omega-3 Fatty Acids

Due to the increasing risks of lifestyle diseases such as hypertension, inflammatory diseases, and diabetes, consumers have become increasingly interested in eggs and meat enriched with omega-3 fatty acids through dietary supplementation with fish oil, linseed products, and microalgae. Several studies proved that the moderate inclusion of fish oil had no detrimental effects on meat physical characteristics [37,50,51,52]. Rahbari et al. [51] noted that the dietary inclusion of 0.057% and 0.5% salmon oil had no negative effect on pH_0h_, pH_4h_, or WHC in breast meat. Likewise, Jeon et al. [50] concluded that dietary 2% salmon oil had no significant impact on pH_24h_ or WHC in thigh meat. Homyok et al. [52] showed that the dietary supplementation of 3% tuna oil had no significant effect on pH_24h_, drip loss, cooking loss, or color (L*, a*, and b* values) in both breast and thigh meats. Elbaz et al. [37] similarly reported that dietary 3% fish oil had no detrimental impact on physical meat characteristics such as pH, cooking loss, and chilling loss in the breast meat of broilers reared under cyclic heat stress conditions (see Section 4.2 for the full comparative findings across oil sources in this study). These results indicated that moderate levels of dietary fish oil had no negative effect on ultimate pH or, consequently, other meat quality characteristics. Regarding linseed oil, Pečjak et al. [53] and El-Bahr et al. [54] observed that dietary inclusion of 4–5% linseed oil had no negative impact on meat physical quality characteristics. Pečjak et al. [53] observed that the dietary supplementation of 5% linseed oil had no significant influence on the pH_15min_, pH_24h_, drip loss, color (L*, a*, and b* values), or electrical conductivity of breast meat. El-Bahr et al. [54] elucidated that the dietary inclusion of 4% linseed oil had no significant effect on pH_24h_, drip loss, thawing loss, cooking loss, the L* value, the a* value, or chroma; however, Warner–Bratzler shear force was decreased in breast meat. Also, Albergamo et al. [55] observed that a dietary mixture of 3.13% extruded linseed meal + 300 mg vitamin E/Kg feed + 0.40 mg Se/Kg feed enhanced juiciness and chewing, reduced drip loss, and had no effect on the pH, color (L*, a*, and b* values), or cooking loss of breast meat. The use of marine algae in chicken feed diets is becoming more popular due to their high content of DHA. Al-Khalaifah et al. [56] documented that the dietary inclusion of *Sargassum* sp. (1% or 2%), *Gracilaria* sp. (0.5% or 1%), and *Spirulina* sp. (5% or 7.5%) had no significant effect on taste, flavor, color, smell, texture, appearance, or overall acceptance. Similarly, Jeon et al. [50] concluded that a 20 g/kg diet of *Schizochytrium* sp. had no negative effect on pH_24h_ or WHC in thigh meat. Khan et al. [57] postulated that dietary 2% DHA-rich microalgae increased drip loss, increased shear force, reduced the a∗ value, reduced the b∗ value, decreased the occurrence of white striping and myopathy, and had no significant impact on pH or cooking loss in breast meat. Taken together, dietary omega-3 fatty acid sources including fish oils and linseed products generally had limited effects on pH and WHC at the inclusion levels tested (≤5%), and most sources showed no consistent effect on color. However, higher-DHA marine algal inclusion (e.g., 2% DHA-rich microalgae) can increase drip loss and shear force while reducing the a* and b* values, indicating that physical trait outcomes for omega-3 enrichment are dose- and source-dependent rather than uniformly neutral, even though such changes were accompanied by a reduced incidence of white striping and myopathy in that study.

**Figure 3:**
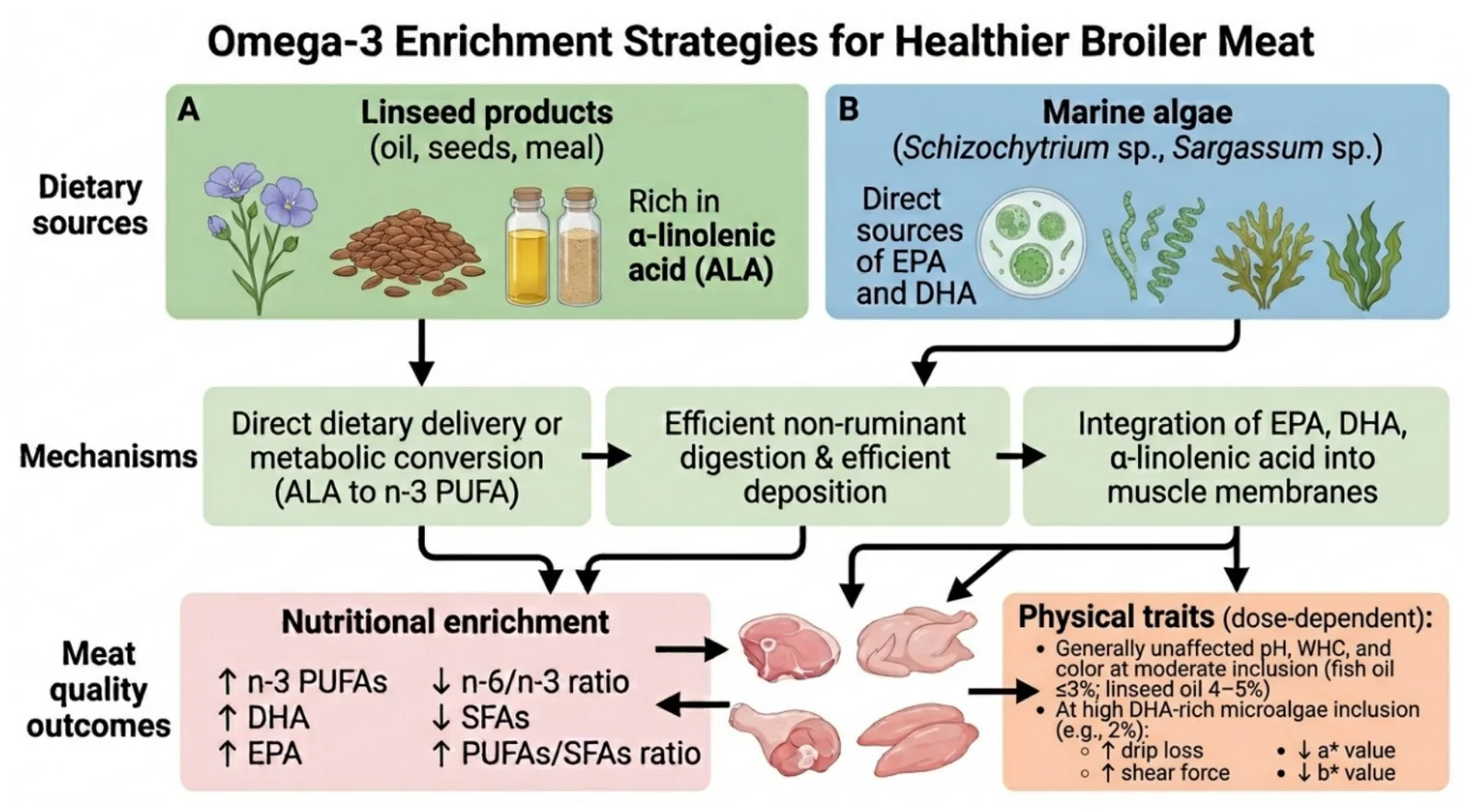
Conceptual framework of omega-3 enrichment strategies in broiler meat (↑: Increase and ↓: Decrease).

Several studies indicated that the dietary inclusion of omega-3 sources (fish oil or linseed oil) is an effective strategy to produce omega-3-enriched meat without adverse effects on the proximate composition of broiler meat [37,50,52,53,54]. Jeon et al. [50] concluded that dietary 2% salmon oil had no significant effect on meat chemical composition (moisture, CP, EE, and crude ash); modified the lipid profile (reduced SFAs, increased UFAs, increased PUFAs, increased PUFA/SFA ratio, reduced n-6/n-3 PUFA ratio); and increased TBARS after 7 days of storage of thigh meat. Also, Homyok et al. [52] showed that the dietary supplementation of 3% tuna oil increased the meat concentrations of n-3PUFAs, n-6PUFAs, and total PUFAs and the n-3/n-6 PUFA ratio; however, it had no significant influence on SFAs or MUFAs in breast meat. Elbaz et al. [37] reported that dietary 3% fish oil increased USFAs, PUFAs, the UFA/SFA ratio, the PUFA/SFA ratio, and the n-3/n-6 PUFA ratio, while it had no significant effect on SFAs or malondialdehyde (MDA) in the breast meat of broilers reared under cyclic heat stress conditions. Similarly, it was observed that the inclusion of 0.057 and 0.5% salmon oil had no significant impact on MDA concentration in breast muscle [51]. Regarding linseed oil, Pečjak et al. [53] observed that the dietary supplementation of 5% linseed oil increased the concentrations of n-3PUFAs and the n-3/n-6 PUFA ratio and decreased SFA and MUFA contents; however, it had no significant effect on the γ-tocopherol content, Se content, or antioxidant capacity of water-soluble antioxidants, while MDA content increased in fresh and chilled breast meat stored at 4 °C for 6 days, likely because PUFA-enriched meat is more susceptible to lipid oxidation. El-Bahr et al. [54] elucidated that the dietary inclusion of 4% linseed oil increased the breast muscle contents of α-linolenic acid (ALA, C18:3 n3), docosahexaenoic acid (DHA, C22:6 n-3), eicosapentaenoic acid (EPA, C20:5 n-3), linoleic acid (C18:2), arachidonic acid (ARA, C20:4 n-6), PUFAs, and n-3PUFAs, while the concentrations of SFAs and MUFAs were decreased. They also indicated that the dietary inclusion of 4% linseed oil increased the breast muscle concentrations of some essential amino acids (arginine, histidine, phenylalanine, and threonine) and some nonessential amino acids (alanine, asparagine, and glutamine). Albergamo et al. [58] observed that a dietary mixture of 3.13% extruded linseed meal + 300 mg vitamin E/Kg feed + 0.40 mg Se/Kg feed increased total PUFAs, n-3 PUFAs, Se, Fe, Mn, Cd, and Pb, while SFAs were reduced in breast meat. Several recent studies proved that marine algae could be utilized as successfully as fish oil or flaxseed oil to increase the percentage of n-3 PUFAs in broiler meat [50,56,57]. *Schizochytrium* sp. is a promising source of n-3PUFAs (especially DHA) and antioxidants. Jeon et al. [50] concluded that a 20 g/kg diet of *Schizochytrium* sp. had no negative effect on meat chemical composition (moisture, CP, EE, or crude ash), increased the concentrations of DHA, reduced the n-6/n-3 PUFA ratio, and minimized TBARS after 7 days of storage of thigh meat. Khan et al. [57] postulated that the dietary inclusion of 2% DHA-rich microalgae increased the n-3PUFA and total long-chain (≥20C) n-3PUFA contents of breast meat, while mineral (Ca, P, Mg, and Na), cholesterol, CP, and TBARS contents were not affected. Al-Khalaifah et al. [56] documented that the dietary inclusion of 1.0 and 2.0% of Sargassum sp. reduced EE content, modified the lipid profile (elevated the concentrations of n-3PUFAs and n-3/n-6 PUFA ratio and decreased MUFA content), enhanced antioxidative properties (increasing SOD and reducing MDA concentrations) and did not affect CP, ash, or moisture in breast meat. It was documented that dietary omega-3 is quickly incorporated into poultry tissues because birds, non-ruminant animals, absorb dietary lipids with minimal biohydrogenation, allowing for the efficient transfer and deposition of them into edible meat [59]. When comparing the three principal omega-3 enrichment strategies—fish oil, linseed products, and marine algae—their relative efficacy depends on the specific n-3 fatty acid targeted, the oxidative stability of the resulting meat, and sensory acceptability. Fish oil and marine microalgae (e.g., *Schizochytrium* sp.) provide the greatest enrichment efficiency for long-chain n-3 PUFAs (EPA and DHA), since these sources supply preformed long-chain fatty acids that are directly incorporated into muscle phospholipids with minimal endogenous biosynthesis required [50,59]. In contrast, linseed (flaxseed) oil supplies predominantly LA, which must be endogenously elongated and desaturated to EPA and DHA; this conversion is inherently inefficient in poultry due to low hepatic Δ6-desaturase and elongase activity, so dietary linseed inclusion substantially raises ALA and total n-3 PUFA content but yields comparatively modest increases in long-chain n-3 derivatives [53,54,58]. Consequently, for applications specifically targeting DHA/EPA enrichment for human health messaging, fish oil and marine algae are the more effective strategies, whereas linseed products represent a lower-cost, more readily available alternative suited to boosting total n-3 PUFA and ALA content without requiring marine-derived ingredients. Collectively, dietary omega-3 fatty acid sources are effective tools for producing omega-3-enriched broiler meat with improved nutritional value and potential health benefits for consumers. Moreover, dietary marine algae provide beneficial antioxidants that help in maintaining meat oxidative stability via reducing lipid peroxidation and consequently increasing the shelf life of such healthy poultry meat (Table 3, Figure 3).

### 4.4. Energy and Enzymes

One of the most recent trends is reducing apparent metabolizable energy (AMEn) in feed or using low-density agro-industrial byproducts and compensating for this by the addition of carbohydrases (β-mannanase, β-glucanase, xylanase, cellulase, pectinase, and amylase), which might be involved in improving their utilization and reducing energy depletion in an efficient way [11]. Several studies demonstrated that the dietary supplementation of β-mannanase or a multi-enzyme cocktail sustained meat color, WHC, tenderness, and cooking loss in broiler meat [60,61,62,63]. Lee et al. [63] noted that reducing AMEn by −100 kcal/kg with the dietary supplementation of β-mannanase (800,000 U/kg) increased the b* value and did not have a significant impact on pH, the L* value, the a* value, WHC, or TBARS in breast meat. Nowadays, there is more interest in multi-enzyme cocktails in poultry diets, since combining enzymes with complementary substrate specificities can achieve synergistic benefits beyond those obtained with single-enzyme supplementation. These synergies arise through several complementary mechanisms: (1) complementary substrate specificity across the non-starch polysaccharide (NSP) fraction, which is involved in releasing entrapped starch, protein, and lipid for enzymatic and endogenous digestion [62]; (2) viscosity reduction effects that indirectly enhance co-administered enzymes’ access to the substrate [60,64]; and (3) the combined liberation of nutrients trapped in the protein–carbohydrate–phytate matrix [65]. Kim et al. [62] reported that decreasing AMEn by −100 kcal/kg plus the addition of 0.1% xylanase alone or a 0.1% xylanase and β-glucanase combination increased pH_24h_, decreased the a* value and did not affect pH_1h_, the L* value, the b* value, or WHC in breast meat. Moreover, Attia et al. [60] pointed out that the dietary inclusion of 0.1 and 0.2% of a commercial multi-enzyme cocktail (Galzym-M, Tex Biosciences, UK; an overall product activity of 100,000,000 U/kg containing cellulase, together with 1,500,000 U/kg of 4-β-xylanase, 6500 U/kg of lipase, 250,000 U/kg of α-amylase, 40,000 U/kg of protease, and 30,000 U/kg of pectinase) in a low-calorie diet (−176 kcal/kg) had no negative impact on meat quality characteristics (pH, color, tenderness, and WHC) in both breast and thigh meat. These findings are in accordance with Bromfield et al. [64] who demonstrated that a low-energy diet (reduced by −150 and −200 kcal/kg) with 350, 700, and 1000 g/ton multi-enzymes added (1500 U/g of phytase,10,000 U/g of xylanase, 6000 U/g of cellulase, 400 U/g of amylase, 700 U/g of protease, 700 U/g of β-glucanase, and 400 U/g of mannanase) had no negative impact on meat quality traits, except for breast pH, which increased significantly when multi-enzymes were added by 350 and 700 g/ton to the low-energy diet (−150 kcal/kg diet). Also, Hussein et al. [65] elucidated that a low-caloric diet (−100 kcal/kg) with the addition of a 0.50 g/kg diet of Kemzyme Plus^®^ (containing multiproteases, multiamylases, and NSP-hydrolyzing enzymes) increased shear force, reduced the b* value, and had no significant effect on the pH_15min_, WHC, L* value, a* value, cooking loss, hardness, springiness, cohesiveness, or chewiness of breast meat. Yaqoob et al. [61] showed that a reduction in AMEn by −50 kcal/kg and the dietary inclusion of 100 mg/kg multi-enzymes (1,4-β-xylanase 20,000 U/g; α-amylase 2000 U/g and protease 40,000 U/g) improved meat pH_24h_ and had no effect on color (L*, a*, and b* values), WHC, cooking loss, or shear force in breast meat. These findings suggest that dietary exogenous enzymes improve nutrient digestibility and may compensate for reduced dietary energy levels while generally preserving pH, WHC, cooking loss, and proximate composition [11,60,64]. Meat color outcomes were largely stable across most enzyme/dose combinations, although isolated exceptions occurred: β-mannanase supplementation increased the b* value in one study [63], and xylanase ± β-glucanase decreased the a* value in another [62]. Texture was likewise mostly unaffected, but Hussein et al. [65] reported increased shear force and a reduced b* value with Kemzyme Plus^®^ supplementation, indicating that not all commercial enzyme preparations produce equivalent outcomes. This might indicate that dietary exogenous carbohydrases and multi-enzyme cocktails generally help maintain broiler meat quality (pH, color, tenderness, and WHC) when dietary energy or nutrient density is reduced. However, their effects are not always consistent, as some studies have reported changes in specific traits such as pH, color, or shear force (Table 4). These differences likely stem from variation in enzyme type, dose, the degree of energy/nutrient reduction, and feed substrate composition, rather than a true contradiction—and the observed changes (e.g., higher pH_24h_) do not necessarily indicate quality impairment. Overall, exogenous carbohydrase and multi-enzyme supplementation generally maintain broiler meat quality, with some dose- and substrate-dependent effects on specific traits (Table 5). Further studies using standardized enzyme preparations and dietary formulations are still needed to clarify the conditions under which enzyme supplementation can alter specific meat quality characteristics.

## 5. Feeding Regimes and Management Influencing Broiler Meat Quality

### 5.1. Feed Restriction Programs

**Figure 4:**
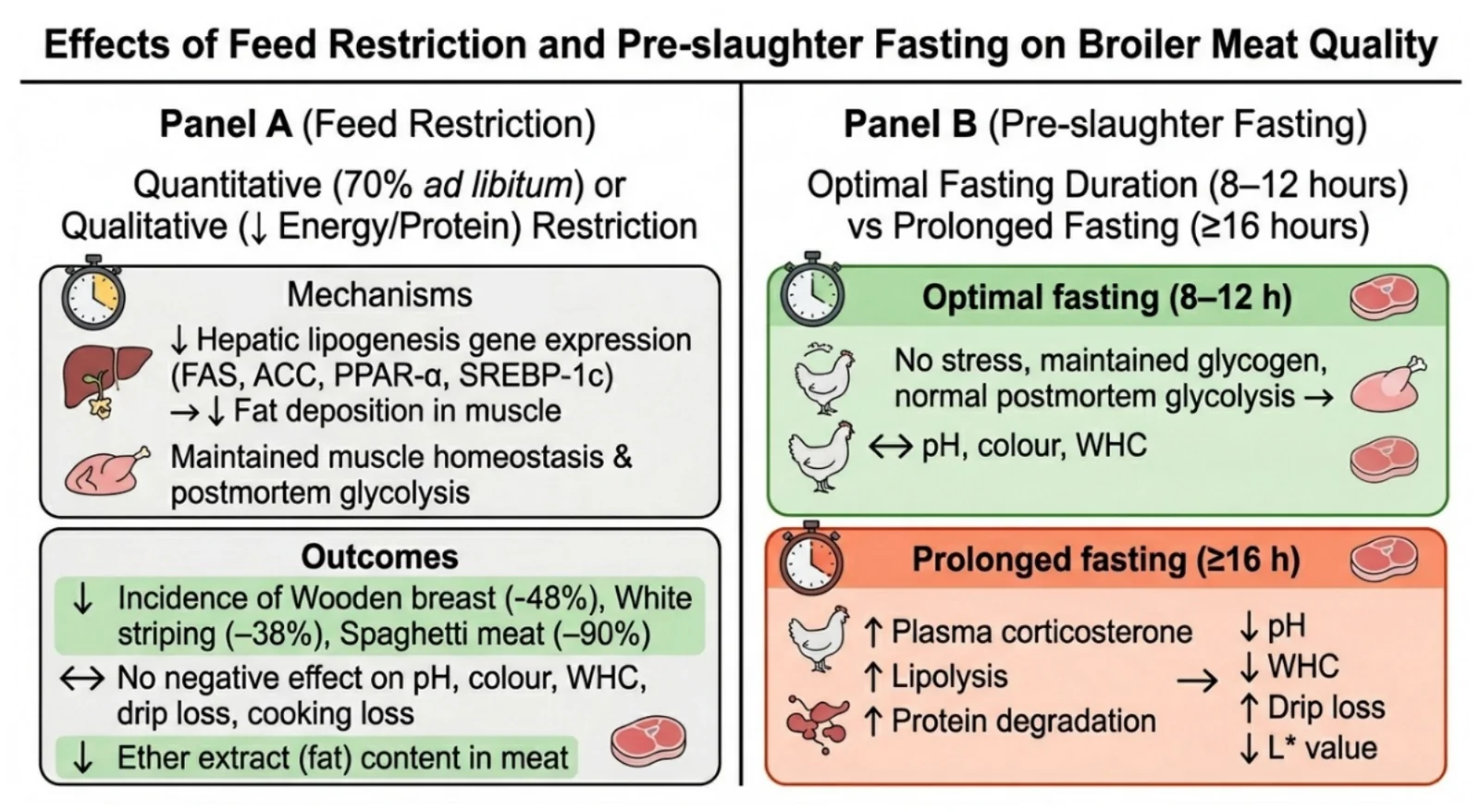
A dual-panel conceptual diagram showing the effects of feed restriction programs (Panel (**A**)) and pre-slaughter fasting (Panel (**B**)) on broiler meat quality (↑: Increase and ↓: Decrease).

Feed restriction programs are divided into quantitative feed restriction (quantity-limited feed or time-limited accessibility to the feeders) and qualitative feed restriction (decrease in nutritional density in particular protein, amino acids, and energy or diet dilution by addition of fibers) [10]. The present section updates and extends this classification framework, originally presented in Ebeid et al. [10], by incorporating primary studies published subsequently and by situating feed restriction outcomes alongside five additional diet manipulation strategies within a single comparative, physicochemically organized framework, rather than as a stand-alone topic. Numerous studies explained that quantitative feed restriction [66,67,68] and qualitative feed restriction [69,70,71] had no negative effect on physical meat quality traits. Regarding quantitative feed restriction, Ye et al. [66] reported that 80% of *ad libitum* intake had no negative effect on pH_45min_, pH_24h_, drip loss, shear force, appearance, odor, tenderness, juiciness, or broth freshness, while the L* value was decreased and the a* value was increased in the *Pectoralis* major muscle of Bearded chickens, a slow-growing indigenous Chinese breed rather than a fast-growing commercial broiler strain. Similarly, Tůmová et al. [68] pointed out that physical meat quality parameters (pH, color, drip loss and cooking loss) were not affected by feed restriction (70% of *ad libitum* intake from 8 to 14 days of age) or feed restriction coupled with access to pasture. Also, Tůmová et al. [67] concluded that quantitative feed restriction (70% of *ad libitum* intake from 14 to 21 d of age) had no significant influence on the pH, meat color (L*, a*, b* values), drip loss, cooking loss or shear force of breast muscle. With respect to qualitative feed restriction, Shen et al. [71] postulated that low-nutrient diets (low metabolizable energy at 11.7 MJ/kg and low protein at 17.0%) had no negative effect on the pH_45min_, meat color (L*, a*, and b* values), drip loss, or cooking loss of both thigh and pectoral muscles. Karaarslan et al. [70] documented that a low-energy (reduced by 10%) and -protein (reduced by 20%) diet had no significant effect on pH_15min_, pH_24h_, pH_72h_, the L* value, the b* value, chroma, or cooking loss, while the a* value increased but the Hue angle was reduced in breast meat. Also, Cui et al. [69] pointed out that low metabolizable energy (2880 kcal ME/kg) did not significantly affect pH (45 min and 24 h), the L* value, drip loss appearance, tenderness, or juiciness; however, shear force was reduced significantly in breast meat in slow-growing yellow-feathered chickens. These results are in harmony with Ahsan and Cengiz [72], who confirmed that meat pH and color (L*, a*, and b* values) were not influenced by a low-nutrient-density diet (5% reduction in metabolizable energy and amino acids, plus 15% reduction in lysine). Within the same study, this qualitative restriction produced two seemingly opposing outcomes: on one hand, the drip loss of the *Pectoralis* major muscle was increased, an unfavorable effect on WHC; on the other hand, the incidence and severity of white striping were reduced, a favorable effect on meat quality. Rather than being contradictory, these two findings likely reflect distinct underlying mechanisms. The increased drip loss may be attributable to slower postmortem muscle growth and reduced myofiber hypertrophy under restricted lysine and energy intake, which can alter sarcomere structure and membrane integrity in ways that modestly compromise water retention. In contrast, the reduction in white striping severity is consistent with the restricted growth rate slowing the mismatch between rapid myofiber hypertrophy and the capacity of the vascular and connective tissue network to support it—a mismatch considered a primary driver of white striping and related myopathies in fast-growing broilers. Moreover, Huerta et al. [18] concluded that dietary fat lessening (7.7% vs. 6.7% in grower diet; 8.9% vs. 7.7% in finisher diet) had no adverse effect on meat quality characteristics including pH, color (a*, b*, and L* values), thawing loss, and cooking loss as well as sensory traits (cohesiveness, hardness, juiciness, chewiness, fibrosity, and flavor), while it reduced the percentages of white striping (−38%), wooden breast (−48%), and spaghetti meat (−90%) in the *Pectoralis* major muscle of broilers. These findings suggest that moderate feed restriction was able to maintain normal muscle physiology and postmortem metabolic activity without compromising meat quality [68]. Thus, feed restriction strategies (quantitative or qualitative) did not negatively affect physical meat quality characteristics in broilers. Feed restriction strategies are effective in reducing breast meat myopathies including wooden breast, white striping, and spaghetti meat (Figure 4).

Several studies explained that feed restriction might have a significant effect on meat chemical composition [67,69,72]. Ye et al. [66] reported that quantitative feed restriction (80% of *ad libitum* intake) increased the muscle contents of threonine, lysine, aspartic acid, glutamic acid, proline, and arginine and the activity of the calpain system (which plays a key role in postmortem tenderization of skeletal muscle) in the breast muscle of Bearded chickens, a slow-growing indigenous Chinese breed rather than a fast-growing commercial broiler strain. Tůmová et al. [68] pointed out that feed restriction (70% of *ad libitum* intake from 8 to 14 days of age) or feed restriction coupled with access to pasture had no significant effect on the meat content of DM, CP, cholesterol, and fatty acids, while EE was reduced. Also, Tůmová et al. [67] elucidated that 70% of *ad libitum* intake from 14 to 21 d of age reduced EE and energy content, while DM, CP, ash, and cholesterol concentrations were not influenced in the *Pectoralis* major muscle. Ye et al. [66] and Tůmová et al. [67,68] observed that feed restriction reduced EE concentrations in chicken muscles. This reduction in EE content in meat was connected with the reduced mRNA expression of important genes in lipid metabolism including fatty acid synthase (*FAS*), acetyl-CoA carboxylase (*ACC*), peroxisome proliferator-activated receptor alpha (*PPAR-α*), and sterol regulatory element-binding protein 1c (*SREBP-1c*) in the liver due to feed restriction [66]. Ahsan and Cengiz [72] demonstrated that 5% low-metabolizable-energy and -amino-acid diets and 15% low-lysine diets decreased CP content in breast meat, whilst EE, DM, and ash contents were not influenced. Cui et al. [69] reported that a low metabolizable energy level (2880 kcal ME/kg) altered the volatile compound profile of broiler meat. Specifically, it reduced the concentrations of several aldehydes (hexanal, heptanal, 2,4-nonadienal, octanal, nonanal, and 2-decenal), alcohols (2-nonen-1-ol, trans-2-undecen-1-ol, 7-hexadecenal, 2-hexyl-1-decanol, and 1,3,7,11-trimethyl-1-dodecanol), an alkane (2,6-dimethylheptadecane), and a carboxylic acid (9-hexadecenoic acid) while increasing the concentrations of octadecanal, octadecane, heneicosane, and tetradecanal. Moreover, Huerta et al. [18] concluded that dietary fat lessening (7.7% vs. 6.7% in grower diet; 8.9% vs. 7.7% in finisher diet) had no negative effect on meat chemical composition, including the moisture, CP, ash, and EE concentrations of the *Pectoralis* major muscles of broilers. This might indicate that feed restriction reduced both the EE and energy content of meat and sometimes modified amino acids and volatile compounds in meat. Feed restriction may play a crucial role in determining the gene expression of lipid metabolism (Table 6).

### 5.2. Pre-Slaughter Fasting (Feed Withdrawal)

Pre-slaughter fasting or feed withdrawal is a usual procedure for minimizing carcass contamination, and its effect on meat quality characteristics is duration-dependent, ranging from negligible at moderate durations to detrimental when prolonged. In broilers, pre-slaughter fasting induces physiological stress that alters metabolism, accelerates muscle protein degradation, and promotes glycogen depletion and glycolysis, leading to the increased accumulation of lactic and phosphoric acids in muscle tissue [73,74]. Katsumata et al. [75] observed that pre-slaughter fasting time (8, 16, or 24 h) resulted in stress (increased plasma corticosterone concentration), increased lipolysis (increased plasma nonesterified fatty acid concentration), increased protein degradation (increased plasma Nτ-methylhistidine concentration), increased glutamic acid content, and increased umami taste and intensity, while MDA concentration was not affected in *Pectoralis* major muscles. Schneider and Gewehr [76] and Gewehr et al. [77] indicated that pre-slaughter feed withdrawal (4–12 h) had no detrimental impacts on physical meat quality characteristics. Schneider and Gewehr [76] reported that different pre-slaughter feed withdrawal durations (4, 8, 12, or 16 h) did not significantly affect the cooking loss or WHC of breast meat in male and female broilers slaughtered at 35 or 42 d of age. However, prolonged fasting, particularly 12–16 h, was associated with greater lipid oxidation in 42-day-old birds, indicating that extended fasting may affect meat oxidative stability despite having no apparent effects on these physicochemical quality traits. Similarly, Gewehr et al. [77] showed that pre-slaughter fasting time (4, 8, 12 and 16 h) had no effect on pH_24h_, color (L*, a*, and b* values), shear force, or lipid oxidation (TBARS content) in the breast meat of broilers males and females. These results indicated that a short pre-slaughter fasting period did not stimulate oxidative stress in breast muscle. Wu et al. [74] investigated different pre-slaughter fasting times (4, 8, 12, 16, or 20 h) in broilers and reported that drip loss, pH_45min_, and pH_24h_ were progressively increased with a prolonged fasting duration, but the L* value, cooking loss, and moisture content in leg meat were progressively decreased. They also noted that shear force was reduced with a longer pre-slaughter fasting time, while the a* and b* values were not affected in breast or leg meat. They concluded that moderate fasting periods (8–12 h) generally maintain acceptable meat quality characteristics in broiler chickens. Erener et al. [78] pointed out that feed withdrawal for 10 h before slaughtering increased the ultimate pH and decreased the L* and a* values in thigh meat; however, the addition of chamomile (50 mL/L) and linden (50 mL/L) extracts to drinking water reduced the adverse impact of feed withdrawal on thigh meat’s L* and a* values, keeping them closer to the levels of *ad libitum* feeding. Xue et al. [79] studied the effect of different periods of pre-slaughter feed withdrawal (4, 6, 8, and 10 h) in broilers and noted that prolonged feed withdrawal seems to decrease pH_45min_ but increase the L* value and drop loss, while pH_24h_, shear force, the a*value, and the b* value were not affected in chest meat. Thus, it might be concluded that optimal pre-slaughter fasting time (8–12 h) had no detrimental effects on meat quality characteristics, while prolonged fasting (≥16 h) might negatively impact pH and other quality attributes (Table 7 and Table 8). Further studies are required to clarify the effect of pre-slaughter feed withdrawal on meat quality at the level of gene expression.

## 6. Conclusions

It might be concluded that the integration of precise diet manipulation and a feeding regime and management contributes meaningfully to maintaining, and in specific cases (e.g., omega-3 enrichment, oxidative stability) improving, broiler meat quality. The present review (Part 1) focused specifically on diet manipulation and feeding management strategies; the companion review (Part 2) will extend this analysis to functional feed additives, providing a complementary perspective on nutritional tools available to the poultry industry for safeguarding and enhancing meat quality. Moderate dietary crude protein reduction (−1.5% to −3.0%), when balanced with essential amino acids, maintains breast meat pH, color, and proximate composition while lowering feed cost and nitrogen excretion, although this review did not evaluate direct economic or environmental data to confirm the magnitude of these benefits. Dietary crude protein reductions exceeding −3.0% without adequate amino acid supplementation instead shift nutrient partitioning toward fat deposition rather than lean tissue accretion. The substitution of dietary lipid sources (vegetable oils, insect fats, byproduct oils, with or without emulsifiers) alters the fatty acid profile of meat according to the fat source used, without compromising pH, color, or cooking loss, and exogenous emulsifiers provide additional benefit primarily under oxidative or metabolic stress conditions. The dietary inclusion of omega-3 sources—fish oil (up to 3%), linseed oil/meal (4–5%), and marine microalgae (0.5–7.5%, depending on species)—consistently increases n-3 PUFA and DHA/EPA deposition and reduces the n-6/n-3 ratio in breast and thigh meat, with marine algae additionally improving oxidative stability (reduced TBARS/MDA) and thereby extending shelf life. These enrichment strategies do not compromise the basic physicochemical properties of meat. The supplementation of carbohydrases or multi-enzyme cocktails (e.g., xylanase, β-mannanase, β-glucanase) allows apparent metabolizable energy to be reduced by 50–200 kcal/kg without adverse effects on meat pH, color, WHC, or proximate composition. Quantitative (70–80% of *ad libitum* intake) and qualitative (reduced energy/protein density) feed restriction programs reduce the incidence of wooden breast, white striping, and spaghetti meat by up to 38–90% and lower ether extract content in muscle, without impairing pH, color, or WHC. A pre-slaughter fasting of 8–12 h preserves meat quality parameters, whereas fasting for ≥16 h increases plasma corticosterone and lipolysis, accelerates protein degradation, and produces measurable declines in pH regulation and WHC. Collectively, these findings indicate that crude protein reduction, dietary lipid source and emulsifier selection, omega-3 enrichment, exogenous enzyme supplementation, feed restriction, and controlled pre-slaughter fasting duration are the six nutritional and management levers with the most direct, quantifiable influence on broiler meat physicochemical and nutritional quality, and each carries a defined effective range beyond which quality benefits are lost or reversed.

## Figures and Tables

**Table 1:** Effects of crude protein and amino acid modulation strategies on broiler meat quality.

Ingredient/Strategy Applied	Main Results	Reference
CP −3% + isoleucine (0.26%), whole fattening period	↓ a* value, ↓ L* value, ↓ drip loss ↑ pH_24h_, ↓ shear force ↓ Fat accumulation via AMPK and JAK2/STAT3 activation	[31]
CP −2.0% + 2.5% meat and bone meal (grower 20.4% CP, finisher 19.5% CP)	↑ pH_24h_, ↑ sarcoplasmic protein solubility ↓ b* value, ↓ cooking loss No change in L* value, a* value, shear force or proximate composition	[32]
Low-CP diets, grower (17.5% vs. 18.7/20.5%) and finisher (16.6% vs. 18.0/19.5%)	↑ L* value, ↑ thawing loss ↑ Myofiber size ↓ Myofiber density	[28]
CP −1.5% to −3.0%, soybean meal partially replacing corn/canola meal	↓ b* value, ↓ cooking loss, ↓ shear force No effect on protein, lipid, or DM content	[29]
CP −1% to −4% (grower/finisher, control 20% CP)	No effect on pH, L* value, b* value, cooking loss, shear force, CP, or ash −1% CP ↑ WHC, ↑ a* value; dose-dependent ↓ moisture, ↑ EE	[30]
CP −1.0% + exogenous protease (100–200 ppm)	No effect on L* value, a* value, cooking loss ↑ b* value	[35]
CP −2.0%	Thigh: ↑ b* value, ↑ L* value, no effect on a* value/pH Breast: No effect on pH, a* value, b* value, L* value	[34]

↑: Increase and ↓: Decrease.

**Table 2:** Effects of fat sources and emulsifier strategies on broiler meat quality.

Ingredient/Strategy Applied	Main Results	Reference
3% oil sources: soybean, corn, olive, or fish oil	No effect on pH, cooking loss, chilling loss Soybean oil specifically ↑ shear force, ↑ a* value, ↓ L* value, ↓ b* value	[37]
6% olive pomace acid oil vs. palm oil/refined olive pomace oil	No negative effect on color or sensory scores ↑ MUFAs, ↑ ALA, ↑ n-3 PUFA ↓ α-tocopherol No effect on TBARS	[38]
5% rice bran oil replacing 5% palm oil	↓ pH_20min_, ↓ pH_24h_ ↑ Cooking loss (breast/thigh) ↓ CP, ↓ n-6/n-3 ratio, ↓ SFAs ↑ EE, ↑ ALA, ↑ n-3PUFAs	[39]
5% soybean oil vs. lard	↓ Drip loss No effect on pH_45min_, pH_24h_, shear force	[40]
Low-fat Tenebrio molitor larva meal (up to 5% vs. 7.5–10%)	≤5%: No harmful effect 7.5–10%: ↓ pH_24h_, ↓ a* value, ↑ L* value, ↑ drip loss, ↑ cooking loss ↓ CP, ↓ EE, ↓ moisture ↑ SFA, ↑ MUFA, ↓ PUFA	[43]
Black soldier fly larva fat vs. soybean oil/other lipid sources	No effect on pH, color, drip loss (≤48 h) ↑ Drip loss after 48h vs. poultry fat/lard/tallow ↑ n-3PUFA, ↑ ALA	[41]
Degummed vs. acid soybean oil ± emulsifier (350 g/ton glyceryl PEG ricinoleate)	Degummed oil: ↑ WHC, ↑ shear force, ↑ b* value, ↓ pH_24h_ Emulsifier addition gave no extra benefit	[44]
Emulsifier mix (0.05% sodium stearoyl-2-lactylate + 0.10% 1,3-diacylglycerol) + low-energy diet (−100 kcal/kg)	No effect on pH, color, WHC, cooking loss, drip loss	[47]
Lipase (0.04%) ± lysolecithin (0.04%) + low-energy diet (−100 kcal/kg)	No effect on pH, cooking loss, drip loss, WHC, or fatty acid profile	[45]
Lecithin supplementation (1 g/kg)	↑ L* value, ↑ drip loss ↓ Shear force No effect on pH, a* value, b* value	[48]
Lysophospholipid emulsifiers (0.5, 1 g/kg) under oxidative stress	↑ pH, ↑ WHC, ↑ tenderness	[46]
Emulsifier blend (phosphatidyl/lysophosphatidyl choline + PEG ricinoleate, 500 g/ton) + low-energy diet (−50 kcal/kg)	No effect on total lipids, α-tocopherol, oleic, linoleic, or α-linolenic acid content	[49]

↑: Increase and ↓: Decrease.

**Table 3:** Effects of omega-3 fatty acid enrichment strategies on broiler meat quality.

Ingredient/Strategy Applied	Main Results	Reference
Salmon oil (0.057, 0.5%)	No effect on pH_0h_, pH_4h_, WHC, or MDA	[51]
Salmon oil (2%)	No effect on pH_24h_, WHC, or proximate composition ↑ UFA/PUFA ↓ SFA, ↓ PUFA/SFA ratio, ↓ n-6/n-3 ratio ↑ TBARS after 7d storage	[50]
*Schizochytrium* sp. (20 g/kg)	No effect on pH_24h_, WHC, or proximate composition ↑ DHA ↓ n-6/n-3 ratio ↓ TBARS after 7d storage	[50]
Tuna oil (3%)	No effect on pH_24h_, drip loss, cooking loss, color, SFA, or MUFA ↑ n-3/n-6PUFA ratio, ↑ total PUFA	[52]
Fish oil (3%)	No effect on pH, cooking loss, chilling loss, SFA, or MDA ↑ USFA, ↑ PUFA, ↑ UFA/SFA ratio, ↑ n-3/n-6 ratio	[37]
Linseed oil (5%)	No effect on pH, drip loss, color, electrical conductivity, γ-tocopherol, Se, or antioxidant capacity ↑ n-3PUFA, ↑ n-3/n-6 ratio ↓ SFA/MUFA ratio ↑ MDA after 6d storage	[53]
Linseed oil (4%)	No effect on pH, drip loss, thawing/cooking loss, L* value, a* value, or chroma ↓ Shear force ↑ ALA, ↑ DHA, ↑ EPA, ↑ PUFA, ↑ n-3PUFA, select amino acids ↓ SFA/MUFA ratio	[54]
Extruded linseed meal (3.13%) + vitamin E (300 mg/kg) + Se (0.40 mg/kg)	↑ Juiciness, ↑ chewing ↓ Drip loss No effect on pH, color, cooking loss ↑ Total PUFA, ↑ n-3PUFA, ↑ Se, ↑ Fe, ↑ Mn ↓ SFA	[55,58]
Marine algae: Sargassum sp. (1–2%), Gracilaria sp. (0.5–1%), Spirulina sp. (5–7.5%)	No effect on sensory acceptance Sargassum: ↓ EE, ↑ n-3PUFA and n-3/n-6 ratio, ↓ MUFA; ↑ SOD, ↓ MDA No effect on CP, ash, moisture	[56]
DHA-rich microalgae (2%)	↑ Drip loss, ↑ shear force ↓ a* value, ↓ b* value ↓ White striping/myopathy incidence No effect on pH, cooking loss, minerals, cholesterol, or TBARS ↑ n-3PUFA	[57]

↑: Increase and ↓: Decrease.

**Table 4:** Effects of reduced energy and exogenous enzyme supplementation strategies on broiler meat quality.

Ingredient/Strategy Applied	Main Results	Reference
AMEn −100 kcal/kg + β-mannanase (800,000 U/kg)	↑ b* value No effect on pH, L* value, a* value, WHC, TBARS	[63]
AMEn −100 kcal/kg + 0.1% xylanase alone or + β-glucanase	↑ pH_24h_ ↓ a* value No effect on pH_1h_, L* value, b* value, WHC	[62]
Multi-enzyme cocktail (0.1, 0.2%: cellulase, xylanase, lipase, amylase, protease, pectinase) + low-calorie diet (−176 kcal/kg)	No negative impact on pH, color, tenderness, WHC, DM, CP, EE, or ash (breast/thigh)	[60]
Low-energy diet (−150, −200 kcal/kg) + multi-enzymes (350–1000 g/ton: phytase, xylanase, cellulase, amylase, protease, β-glucanase, mannanase)	No negative impact on meat quality traits, except ↑ breast pH at 350–700 g/ton with the −150 kcal/kg diet	[64]
Low-caloric diet (−100 kcal/kg) + Kemzyme Plus^®^ (0.50 g/kg; multiproteases, multiamylases, NSP-hydrolyzing enzymes)	↑ Shear force ↓ b* value No effect on pH_15min_, WHC, L* value, a* value, cooking loss, or texture parameters	[65]
AMEn −50 kcal/kg + multi-enzymes (100 mg/kg: xylanase, amylase, protease)	↑ pH_24h_ No effect on color, WHC, cooking loss, shear force	[61]

↑: Increase and ↓: Decrease.

**Table 5:** Summary of effects of diet manipulation strategies on meat quality traits in broilers.

Nutritional Strategy	Main Impacts on Meat Quality	Nutritional/Physiological Interpretation	References
**Reduced crude protein diets (−1.5% to −3.0%, amino-acid-balanced)**	Improved pH or unchanged stability. Reduced drip and cooking losses when amino acids were fully balanced. Increased thawing loss reported when CP reduction was not fully compensated by amino acid supplementation. Minimal effects on meat chemical composition at moderate reduction levels; excessive reduction (>3%) shifts nutrient partitioning toward lipid deposition.	Balanced amino acid supplementation may preserve muscle protein deposition and reduce excessive fat accumulation, but the magnitude and direction of physical trait changes depend on the degree of CP reduction and amino acid adequacy.	[28,29,31,32,36]
**Lipid source manipulation (fish oil, rice bran oil, insect fat)**	Modified fatty acid profile toward higher PUFAs and n-3 fatty acids. Variable effects on pH and cooking loss.	Dietary fatty acids are efficiently incorporated into muscle lipids and alter membrane composition.	[37,39,41]
**Marine and algal-derived feed ingredients**	Enhanced n-3 PUFA content. Enhanced oxidative stability. No adverse effects on sensory attributes (taste, flavor, texture, and overall acceptability).	Marine bioactive compounds and long-chain omega-3 fatty acids improve nutritional quality and antioxidant protection.	[50,56]
**Reduced apparent metabolizable energy with addition of exogenous enzymes**	Maintained major physical meat quality traits (pH, WHC, cooking loss, tenderness) under reduced-energy diets, with occasional minor shifts in specific color parameters (e.g., b* or a* value) depending on enzyme type and the magnitude of energy reduction.	Improved nutrient digestibility and reduced intestinal viscosity may spare metabolic energy and support muscle development without impairing postmortem muscle metabolism.	[62,63]

**Table 6:** Effects of feed restriction programs (quantitative and qualitative) on broiler meat quality.

Ingredient/Strategy Applied	Main Results	Reference
Quantitative restriction, 80% of ad libitum intake	No effect on pH, drip loss, shear force, or sensory traits ↓ L* value ↑ a* value ↑ Threonine, lysine, glutamic/aspartic acid, arginine ↑ Calpain activity	[66]
Quantitative restriction, 70% ad libitum (8–14 d) ± pasture access	No effect on pH, color, drip/cooking loss, DM, CP, cholesterol, or fatty acids ↓ EE	[68]
Quantitative restriction, 70% ad libitum (14–21 d)	No effect on pH, color, drip/cooking loss, shear force, DM, CP, ash, or cholesterol ↓ EE, ↓ energy content	[67]
Qualitative restriction: low ME (11.7 MJ/kg) + low CP (17.0%)	No effect on pH, color, drip loss, or cooking loss (thigh and pectoral muscle)	[71]
Qualitative restriction: energy −10%, protein −20%	No effect on pH, L* value, b* value, chroma, or cooking loss ↑ a* value ↓ Hue angle	[70]
Qualitative restriction: low ME (2880 kcal ME/kg)	No effect on pH, L* value, drip loss, or sensory traits ↓ Shear force Altered volatile compound profile	[69]
Low-density diet (5% low ME/amino acids; 15% low lysine)	No effect on pH or color ↑ Drip loss ↓ White striping incidence/severity ↓ CP No effect on EE, DM, ash	[72]
Dietary fat reduction (7.7→6.7% grower; 8.9→7.7% finisher)	No effect on pH, color, thawing/cooking loss, or sensory traits ↓ White striping (−38%), wooden breast (−48%), spaghetti meat (−90%)	[18]

↑: Increase and ↓: Decrease.

**Table 7:** Effects of pre-slaughter fasting (feed withdrawal) duration on broiler meat quality.

Ingredient/Strategy Applied	Main Results	Reference
Fasting for 8, 16, or 24 h	↑ Plasma corticosterone ↑ Lipolysis (NEFA) ↑ Protein degradation (Nτ-methylhistidine) ↑ Glutamic acid/umami intensity No effect on MDA	[75]
Feed withdrawal for 4, 8, 12, or 16 h	No detrimental effect on cooking loss or WHC (males/females, 35 and 42 d)	[76]
Fasting for 4, 8, 12, or 16 h	No effect on pH_24h_, color, shear force, or TBARS	[77]
Fasting for 4, 8, 12, 16, or 20 h	↑ Drip loss, ↑ pH_45min_, ↑ pH_24h_ progressively ↓ L* value, cooking loss, moisture progressively ↓ Shear force with longer fasting 8–12 h identified as optimal	[74]
Feed withdrawal for 10 h ± chamomile/linden extracts in drinking water	↑ Ultimate pH ↓ L* value, ↓ a* value (thigh) Herbal extracts mitigated the L* value/a* value decline	[78]
Feed withdrawal for 4, 6, 8, or 10 h	↓ pH_45min_ ↑ L* value, ↑ drip loss with prolongation No effect on pH_24h_, shear force, a* value, or b* value	[79]

↑: Increase and ↓: Decrease.

**Table 8:** Summary of effects of feeding management strategies on meat quality traits in broilers.

Feeding Management Strategy	Main Impacts on Meat Quality	Proposed Physiological Interpretation	References
**Quantitative feed restriction (70–80% ad libitum)**	pH and cooking loss generally unaffected across protocols. Color response varied: L* decreased and a* increased at 80% ad libitum [66], but no color change at 70% ad libitum [67,68]. Reduced EE/fat deposition consistently reported; amino acid and calpain system activity increased in one study [66].	Adaptive metabolic responses, including altered hepatic lipogenic gene expression, may maintain muscle integrity and postmortem glycolysis under moderate restriction, though restriction level and timing influence color outcomes	[66,67,68]
**Qualitative feed restriction (reduced energy/protein density)**	pH and color generally maintained, though a* value increased and Hue angle decreased in one protocol. Shear force was reduced under low-ME diets. Drip loss increased under low-lysine/low-ME diets. Reduced breast myopathies (white striping, wooden breast, spaghetti meat) consistently reported. EE and volatile compound profile altered in some protocols; CP reduced without affecting EE/DM/ash in another.	Reduced dietary energy and fat may decrease excessive muscle growth and metabolic stress, but the specific physical and chemical composition response depends on which nutrients are restricted and by how much	[18,69,70,71,72]
**Pre-slaughter fasting**	Moderate fasting had limited adverse effects, whereas prolonged fasting altered pH decline and water retention.	Glycogen depletion, stress response, and accelerated postmortem metabolism influence meat quality outcomes	[74,77]

## Data Availability

The original contributions presented in this study are included in the article. Further inquiries can be directed to the corresponding author.
